# The impact of Covid-19 pandemic related lockdown on clubfoot practice: Type of study design: Retrospective cross-sectional study

**DOI:** 10.1097/MD.0000000000026389

**Published:** 2021-06-25

**Authors:** Hakan Özbay, Serdar Toy, Oktay Polat

**Affiliations:** Department of Orthopedics and Traumatology, Ağri Training and Research Hospital, Ağri, Turkey.

**Keywords:** clubfoot, COVID-19, pediatrics

## Abstract

We investigated whether the number of pediatric patients with congenital clubfoot treated with the Ponseti method decreased during the Covid-19 pandemic or not in a rural area. So we aimed to guide orthopedic surgeons and health infrastructure for future pandemics to be prepared in hospitals of rural areas for the treatment of children with congenital clubfoot.

One hundred and fifty-four patients with clubfoot who were admitted to our clinic were evaluated retrospectively from March 2017 to December 2020. Institutional hospital electronic database was used to detect the number of weeks between the birth and first cast performed in clinic and the number of casts been applied and unilaterality or bilaterality. Patients were divided into four groups, which included pandemic period and three previous years. Recorded data were analyzed statistically to detect if there is a difference between the numbers of the patients in pandemic period and three previous years.

The number of patients with clubfoot admitted to our hospital between March 2020 and December 2020 increased by 140% compared to previous year. There was a statistically significant difference between the average number of cast applications of Group 4 and other groups (*P* <.001). Achilles tenotomy was performed in 44 (61.1%) of 72 patients admitted during the pandemic period. Only 4 (13.3%) out of 30 patients admitted between March 2019 and December 2019 were performed Achilles tenotomy.

We detected an increase in the number of clubfoot cases admitted to our rural-based hospital during the Covid-19 pandemic, treated with casting or surgically. We think this is because of preventive measures during the pandemic, which caused parents could not reach urban for treatment.

## 1 Introduction

**Figure 1:**
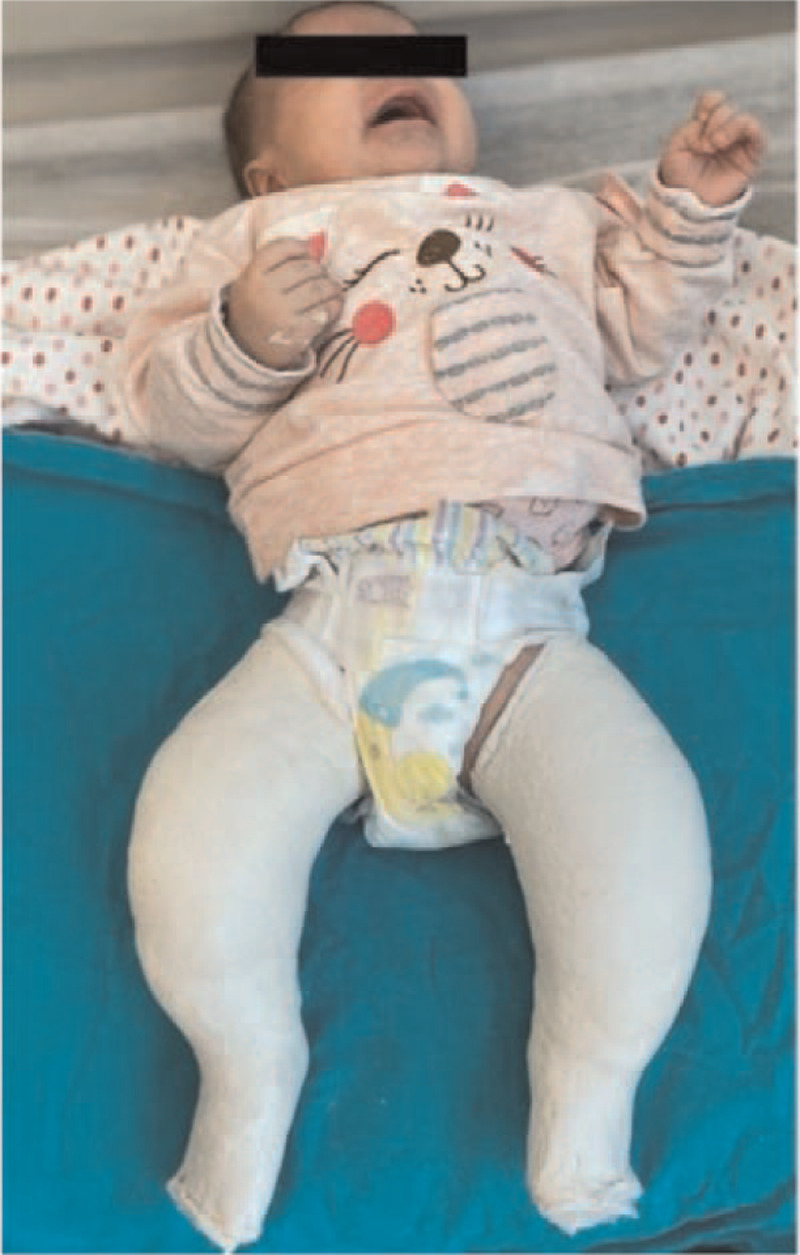
A 2-months old patient with bilateral club foot after the fourth cast in outpatient clinic.

**Figure 2:**
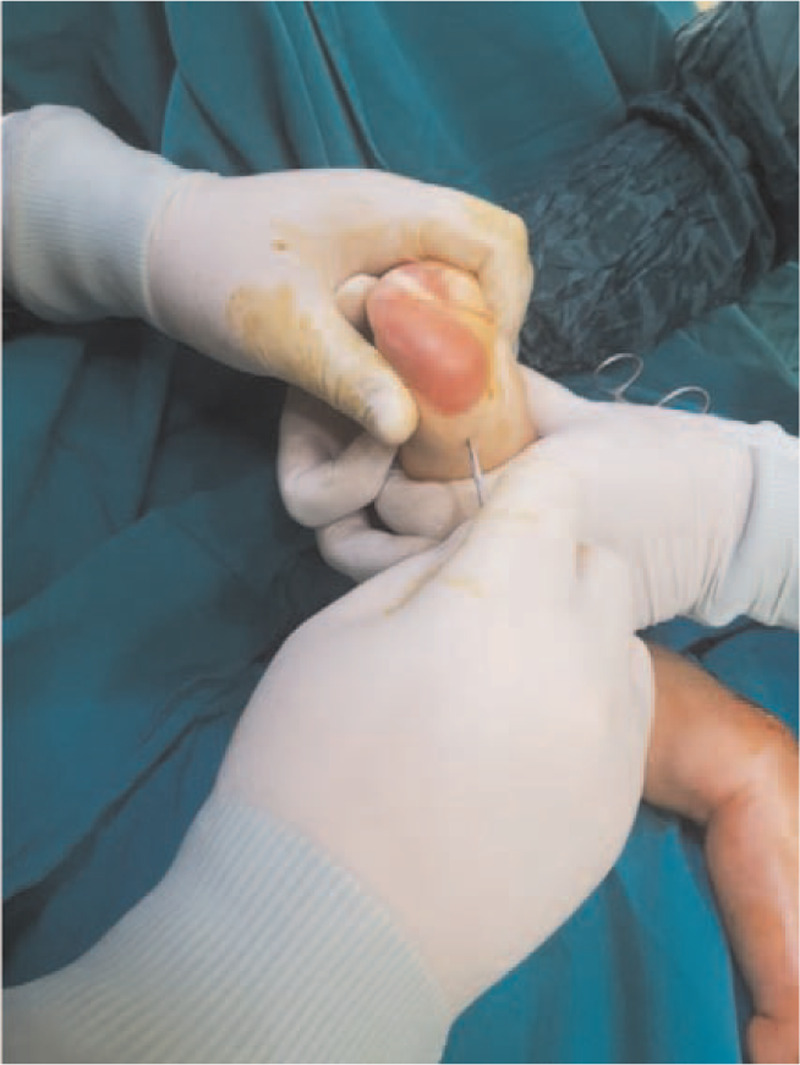
Percutaneous Achilles tenotomy of a 2 months old patient with clubfoot in the operating room.

Congenital clubfoot is the most common pediatric foot disease, which occurs once every 1000 births.^[1,2]^ Although clubfoot is recognizable at birth, the severity of the deformity can range from mild to extremely stiff feet resistant to manipulation. When these patients are not treated in time, they walk on the lateral or tips of their feet, resulting in callus formation, potential skin and bone infections, inability to wear standard shoes, and significant mobility restrictions. The Ponseti method, which is the gold standard in clubfoot treatment, is provided with weekly corrective casts **(**Fig. 1**)**, followed by Achilles tenotomy **(**Fig. 2**)** to correct the equinus deformity, and a long-term abduction orthosis is used to continue the correction.^[1]^

In December 2019, coronavirus disease (COVID-19) emerged in Wuhan city of China.^[3]^ It was later declared as a pandemic by World Health Organization (WHO) in March 2020, spreading all over the world. The virus was highly infectious and had a high mortality rate. Public health measures such as isolation, social distance, and curfews have been declared worldwide as in our country to prevent the spread of the virus and eliminate the damage it causes to the body.^[4]^

The intercity transportation restrictions and the postponement of elective practices during the pandemic period made the treatment of many diseases difficult. It also created difficulties in the Ponseti method, which is considered an elective practice. When we investigated the literature, it was stated that there was a decrease in the number of admissions for clubfoot to the third-level hospitals.^[5–7]^

This study aims to investigate the effect of the pandemic period on the clubfoot treatment retrospectively by evaluating the patients with clubfoot who were treated with the Ponseti method in our clinic before and during the pandemic period and to guide health infrastructure for future pandemics. Our hypothesis in this study is that if the number of patients with congenital clubfoot is increasing during pandemic related lockdown period in hospitals of rural areas, then orthopedic surgeons in these areas should be prepared to diagnose and treat these patients without need of pediatric orthopedic specialists.

## 2 Methods

Our study was performed with 154 patients retrospectively. The patients with clubfoot who were admitted to orthopedics and traumatology outpatient clinic of our hospital were evaluated from March 2017 to December 2020 using institutional hospital electronic database. Inclusion criteria were patients aged between 0 to six months with idiopathic clubfoot and patients with only clubfoot as a congenital malformation cast firstly in our hospital. Exclusion criteria were patients older than six months, patients with neuromuscular diseases or syndromes (secondary cases) and cast first in another hospital and admission date before March each year. Secondary clubfoot cases could not be resumed to treatment because of the need for advanced research in bigger centers. We excluded patients who seek care in January and February, because pandemics emerged in March and we aimed to standardize periods evaluated in this study. We resumed to serve patients who were admitted to our outpatient clinic with traumatic or non-traumatic causes including clubfoot as pandemic spreads over the country. So tenotomy surgeries or weekly casting for clubfoot patients were not interrupted in our hospital. Three different surgeons continued to perform casting weekly in outpatient clinic and tenotomies in operating room, protecting patients and themselves from infection. Our research was conducted in accordance with the principles outlined in the Helsinki Declaration 2008. The study's ethics approval was obtained from our hospital's Clinical Research Ethics Committee (approval date-number: 21.12.2020–31).

Patients were divided into four groups. The first group (Group 1) included patients who were treated in our clinic between March 2017 and December 2017. The second and third groups (Groups 3 and 4) were patients admitted between March 2018 and December 2018, and between March 2019 and December 2019, respectively. The fourth group (Group 4) included patients admitted to our clinic between March 2020 that is when Covid-19 disease was declared a pandemic by WHO and December 2020. The number of weeks between the birth and first cast in clinic, how many cast has been applied and unilaterality or bilaterality were recorded.

### 2.1 Statistical analysis

Demographic data and others were reported as frequency and percentages. The arithmetic means ± standard deviation was calculated for the numerical variables. The distribution characteristics of the data were determined by the Shapiro–Wilk test, while Levene's test calculated the homogeneity of variances. Statistical analysis was performed using SPSS for Windows 23.0 (IBM Corp., Armonk, NY, USA). The comparison between the groups was performed using the ANOVA test. *P* < .05 was considered statistically significant. STROBE checklist was used as a guide in the design of the study.

## 3 Results

The average age of the patients at the time of the first cast was shown in Table 1. Gender, laterality and the number of patients in all groups was detailed in Table 2. While 56 (36.4%) of the patients were performed Achilles tenotomy, it is not known whether it was performed in 98 (63.6%) of patients because they did not continue their follow-up. So patients who were performed with Achilles tenotomy in other centers or not at all and, who were not admitted to our clinic after casting was shown as “unknown” in Table 2.

**Table 1:** Comparison of the mean ages and the number of casts between groups.

		Groups					
		Group 1	Group 2	Group 3	Group 4	Total	*P*-value
Age (weeks)	Mean ± SD	11.14 ± 10.22	4.17 ± 5.54	10.40 ± 10.96	4.56 ± 6.85	6.83 ± 8.78	<.001^∗^
	Range	1-27	1-21	1-29	1-28	1-29	
Number of Cast	Mean ± SD	2.36 ± 1.90	2.75 ± 2.25	3.00 ± 2.85	4.92 ± 3.36	3.74 ± 3.07	<.001^∗^
	Range	1–7	1–7	1–10	1–11	1–11	

SD = standard deviation.

∗ ANOVA test.

**Table 2:** Comparison of gender, laterality and tenotomy if performed between groups.

		Groups					
		Group 1	Group 2	Group 3	Group 4	Total	
		Count N (%)	Count N (%)	Count N (%)	Count N (%)	Count N (%)	*P*-value
Laterality	Unilateral	16 (57.1%)	16 (66.7%)	12 (40.0%)	12 (16.7%)	56 (36.4%)	<.001^∗^
	Bilateral	12 (42.9%)	8 (33.3%)	18 (60.0%)	60 (83.3%)	98 (63.6%)	
	Total	28 (18.2%)	24 (15.6%)	30 (19.5%)	72 (46.8%)	154 (100.0%)	
Achilles tenotomy	Unknown	26 (92.9%)	18 (75.0%)	26 (86.7%)	28 (38.9%)	98 (63.6%)	<.001^∗^
	Done	2 (7.1%)	6 (25.0%)	4 (13.3%)	44 (61.1%)	56 (36.4%)	
	Total	28 (18.2%)	24 (15.6%)	30 (19.5%)	72 (46.8%)	154 (100.0%)	
Gender	Female	12 (42.9%)	10 (41.7%)	12 (40.0%)	38 (52.8%)	72 (46.8%)	.575
	Male	16 (57.1%)	14 (58.3%)	18 (60.0%)	34 (47.2%)	82 (53.2%)	
	Total	28 (18.2%	24 (15.6%)	30 (19.5%)	72 (46.8%)	154 (100.0%)	

N = number.

∗ ANOVA test.

It was determined that clubfoot patients who applied to our hospital between March 2020 and December 2020 continued their follow-up at our hospital for a longer period. An average of 4.92 plaster cast was applied to the patients who applied on these dates. There was a statistically significant difference between the average number of cast applications of Group 4 and other groups (*P* < .001) (Table 1).

The number of patients with clubfoot admitted to our hospital between March 2020 and December 2020 increased by 140% compared to those admitted between March 2019 and December 2019. There was a significant difference between the number of patients with clubfoot admitted in pandemic and other periods (*P* < .001) (Table 2).

Achilles tenotomy was performed in 44 (61.1%) of 72 patients admitted between March 2020 and December 2020. Only 4 (13.3%) out of 30 patients admitted between March 2019 and December 2019 were performed Achilles tenotomy in our hospital, and the remaining patients did not continue their follow-up. There was a significant difference between the number of patients who were performed Achilles tenotomy in pandemic period and other periods (*P* < .001) (Table 2).

## 4 Discussion

An increase in the number of congenital clubfoot cases admitted to our hospital in a rural area during Covid-19 pandemic was detected in this study. So, orthopedic surgeons and health care providers should be informed about increasing trend in the number of these patients during pandemic period to expand their knowledge about diagnosis and treatment of congenital clubfoot disease.

The first Covid-19 case was reported in Turkey in March 2020 after it emerged and spread worldwide. This highly contagious disease is still spreading worldwide despite all preventive measures taken by governments or the public itself. It affected people not to admit a hospital unless there is a severe health problem. Elective surgeries were also postponed to decrease spreading by cross-infection and improve resources and health infrastructure.^[8]^ Bram et al ^[9]^ detected a nearly 60% reduction in total pediatric fracture volume during pandemic compared to prior years. Garcia et al ^[6]^ found a decrease in the number of pediatric trauma cases, the number of patients in outpatient clinics and the number of elective cases compared to other years in their third level pediatric hospital. Covid-19 pandemic has also a significant impact on orthopedic surgery and the practice of foot and ankle specialist surgeons.^[10]^ Our hospital is a rural-based, third-level hospital in Turkey's peripheral region dealing with both Covid-19 and orthopedic patients. We continued to treat orthopedic trauma and pediatric orthopedic patients in emergency department and outpatient clinic during Covid-19 pandemic.

The overall reduction in the number of pediatric cases admitted to outpatient clinics caused a delay in diagnosis and treatment during pandemic because of “stay home” initiatives, fear of people to exist in a crowded environment or lockdown measures implemented by governments. Some guidelines suggested delaying clubfoot treatment (initial management, including tenotomies) by up to 3 months.^[11]^ The United Kingdom Clubfoot Consensus Group offered to postpone clubfoot treatment for the duration of the pandemic in their country.^[12]^ There was no consensus or suggestions about the treatment of the clubfoot except cessation of elective surgeries in our country. So we continued to treat clubfoot patients, casting them weekly.

The treatment of clubfoot with the Ponseti method has also been interrupted because of reasons mentioned. Aroojis et al ^[5]^ stated that pandemic disrupted clubfoot treatment services as patients could not travel to their clinic in their children's hospital in a rural area. They also detected that new patients were not registered, and all casting and bracing visits were suspended. Rangasamy et al ^[13]^ detected a significant reduction in number of their clubfoot cases during the pandemic in their survey among orthopedic specialists. And they noticed a considerable number of orthopedic specialists avoided Achilles tenotomy during the outbreak. We detected a 140% increase in our clubfoot cases during the pandemic compared to the previous year. We also found a significant increase in the number of Achilles tenotomies for these patients. But still we detected that the tenotomy rate in our study was lower than reported in the literature.^[14]^ We think that this difference is associated with patients who missing out the follow-up, not our preference for treatment during the pandemic. Given results, more patients with clubfoot were admitted to our outpatient clinic, and more Achilles tenotomies were performed for these patients compared to the previous year. These results are not consistent with the existing literature. We think we encountered more clubfoot cases because of preventive measures during pandemic, which caused parents could not reach bigger centres for the treatment of their children. Our hospital is in a rural area, and people routinely choose urban-based, bigger centres incorporate pediatric orthopedic specialists. Because of measures taken for Covid-19 pandemics like intercity travel ban or curfew, parents admitted to the nearest orthopedic centre like ours. We also think that other possible reason for our results could be the increase in the number of the births in rural hospitals because of mentioned transportation bans between cities. So, pediatric orthopedic cases like clubfoot decreased in urban-based hospitals but increased in rural hospitals. Although treatment of the pediatric cases like clubfoot is still shifting to pediatric orthopedic specialists in orthopedic practice, we think that every orthopedic surgeon should know and treat these cases to be prepared for extraordinary circumstances like future pandemics.

There are some limitations to our study. Because of its retrospective design, we could not question the exact reasons for patients to choose our hospital during pandemic. We could not detect and evaluate the type of treated clubfoot cases according to Pirani or Dimeglio classification because of missing data and retrospective design of this study. It is also not a multi-centre study, and it limits our study to be applied to the general population. However, more studies with a higher number of patients should be done to yield more realistic results.

## 5 Conclusion

In conclusion, in this study, we found an increase in the number of clubfoot cases admitted to our rural-based hospital during Covid-19 pandemic and treated with casting or surgically. We believe that this study would guide health care providers or orthopedic surgeons in rural area for the treatment of clubfoot cases for future pandemics.

## Author contributions

**Conceptualization:** Hakan Özbay.

**Data curation:** Hakan Özbay.

**Formal analysis:** Serdar Toy.

**Funding acquisition:** Serdar Toy.

**Investigation:** Serdar Toy.

**Methodology:** Serdar Toy.

**Project administration:** Oktay Polat.

**Resources:** Oktay Polat.

**Software:** Oktay Polat.

**Supervision:** Oktay Polat.

**Validation:** Hakan Özbay.

**Visualization:** Hakan Özbay.

**Writing – original draft:** Hakan Özbay.

**Writing – review & editing:** Hakan Özbay.
